# 3D tooth identification for forensic dentistry using deep learning

**DOI:** 10.1186/s12903-025-06017-y

**Published:** 2025-04-30

**Authors:** Hamza Mouncif, Amine Kassimi, Thierry Bertin Gardelle, Hamid Tairi, Jamal Riffi

**Affiliations:** 1 LISAC Laboratory, Department of Informatics, Faculty of Sciences Dhar El Mahraz, Sidi Mohamed Ben Abdellah University, Fez, Morocco; 2 3D Smart Factory, Mohamadia, Morocco

**Keywords:** 3D mesh processing, Teeth classification, Dental identification, Forensic dentistry

## Abstract

The classification of intraoral teeth structures is a critical component in modern dental analysis and forensic dentistry. Traditional methods, relying on 2D imaging, often suffer from limitations in accuracy and comprehensiveness due to the complex three-dimensional (3D) nature of dental anatomy. Although 3D imaging introduces the third dimension, offering a more comprehensive view, it also introduces additional challenges due to the irregular nature of the data. Our proposed approach addresses these issues with a novel method that extracts critical representative features from 3D tooth models and transforms them into a 2D image format suitable for detailed analysis. The 2D images are subsequently processed using a recurrent neural network (RNN) architecture, which effectively detects complex patterns essential for accurate classification, while its capability to manage sequential data is further augmented by fully connected layers specifically designed for this purpose. This innovative approach improves accuracy and diagnostic efficiency by reducing manual analysis and speeding up processing time, overcoming the challenges of 3D data irregularity and leveraging its detailed representation, thereby setting a new standard in dental identification.

## Introduction

Recognition of individual human teeth has long been a cornerstone of the dental curriculum [1]. Proficiency in morphological tooth identification is fundamental for dental practitioners across various clinical procedures and throughout their professional careers. Moreover, the ability to identify isolated human teeth holds significant relevance in forensic odontology and archaeology. During catastrophic events such as natural disasters, plane crashes, fires, or situations involving corpse decomposition and disfigurement, conventional identification methods like fingerprints and iris scans may prove inadequate due to their low resistance. In such scenarios, teeth, known for their resilience to extreme conditions, serve as the most reliable means of human identification. During the 2004 Thailand tsunami, dental evidence was instrumental in identifying 79% of the victims. Similarly, in the 2007 tsunami disaster in India, dental records were the primary means of identification in most cases, surpassing fingerprints and DNA evidence [2]. Moreover, much of the current research that explores the determination of ancestry, sex, and age from teeth has primarily focused on single teeth [3]. In archaeological and anthropological studies, isolated teeth have also been significant for identification purposes. Lately, digital technologies have become increasingly popular in dental practices, providing significant benefits for both dentists and their patients. Tools like digital scanners, cone beam computed tomography (CBCT), and intraoral scanners are now generating a wealth of data, which is helping to streamline clinical processes and improve patient care. Also, the emergence of deep learning approaches and convolutional neural networks made breakthroughs in the field of medical diagnosis [4]. In dental diagnosis, many studies have explored the use of deep learning to classify different types of teeth from images. A significant number of these studies have focused on analyzing entire dentitions, especially through the segmentation and classification of teeth seen in Cone-beam Computed Tomography (CBCT) images [5–7] or intraoral scans [8, 9].

Iftinca et al. [10] proposed an approach utilizing convolutional neural networks for tooth classification. They processed grayscale normalized 2D images using a CNN, optimized with the Adam optimizer and sparse categorical cross-entropy loss function. Similarly Miki et al. [11] employed the AlexNet [12] architecture to identify tooth categories from grayscale images. Using images with dimensions of 227x227 pixels, their approach processes the images through AlexNet to classify the tooth type, this method leverages AlexNet’s powerful feature extraction capabilities to achieve effective dental image analysis. Subsequently, Li et al. [1] proposed a method for identifying tooth categories using 2D images captured from cone-beam computed tomography (CBCT) scans, their approach processes 64x64 pixel tooth images with a seven-layer deep convolutional neural network (CNN) that includes global average pooling and is followed by two fully connected layers, this method was tested on a dataset with four classes: molar, premolar, canine, and incisor, yielding promising results. In contrast to 2D image-based approaches, Tian et al. [13] introduced a novel approach for classifying tooth types on 3D tooth models, they developed a two-level hierarchical architecture based on sparse voxel octrees and 3D CNNs, the first level consists of an Octree convolutional neural network with an encoder style, responsible for extracting features that differentiate various tooth types, the second level comprises fully connected layers trained to predict tooth type using the features provided by the first level, this method leverages the spatial complexity of 3D models for improved classification. Subsequently, Li et al. [14] also developed a technique for identifying teeth based on multiple photographs, they captured six 2D photographs from different angles of a tooth and fed them into a feature extractor encoder using ConvNeXt-S [15] as the backbone, the extracted feature map is then processed by an attention encoder to predict the tooth category, this multi-angle approach enhances the model’s ability to accurately classify teeth based on varied perspectives. However, published research focusing on the identification of isolated teeth, particularly in 3D, remains scarce in the field. Also identifying isolated teeth based on their appearance is challenging due to the anatomical similarities between different tooth categories, which necessitates a high level of expertise.

While current state-of-the-art methods in 3D teeth classification have made significant strides, they often exhibit limitations that hinder their applicability and accuracy. Methods relying solely on 2D images, such as those by Li et al. [14] and Miki et al. [11], are restricted in their ability to capture the full spatial complexity of dental structures. These approaches may struggle with variations in viewpoint and lighting conditions, affecting classification accuracy. Additionally, techniques process 3D objects [13] can be computationally intensive and require substantial data preprocessing, potentially limiting scalability and real-time application. In contrast, our proposed approach addresses these challenges by leveraging a hybrid method that converts 3D tooth meshes into 2D representations for efficient processing by recurrent neural networks. This method not only enhances classification accuracy across diverse datasets but also improves computational efficiency, making it suitable for real-world applications in dental diagnostics and forensic dentistry.

The remainder of this paper is structured as follows: The proposed approach is comprehensively outlined, detailing the process of converting 3D meshes into 2D images and the subsequent classification pipeline using RNNs and fully connected layers, Our chosen 3D representation method for tooth modeling is also elaborated upon, emphasizing its role in capturing detailed anatomical features. Subsequently, the section then describes the dataset utilized, including used instances of the dataset. Results Section presents the experimental outcomes of our approach, comparing its performance against existing methods, followed by a discussion of the impact of different types of network and combination of features on the accuracy and robustness of the architecture classification. Finally, The conclusion summarizes the findings.

## Method

### Triangular mesh

3D representations are essential for capturing the intricate details of objects in a three-dimensional space, offering a comprehensive view that surpasses 2D images. Common types of 3D representations include point clouds, volumetric grids, and 3D meshes. Point clouds consist of a set of data points in space, providing a discrete sampling of the object’s surface but often lacking in connectivity information [16–18]. Volumetric grids, or voxel representations, divide the space into a regular grid of 3D pixels (voxels) [19–21], which can model internal structures but are computationally intensive due to their high memory requirements [22, 23]. Within these, 3D triangular meshes stand out for their efficiency and precision.

**Fig. 1:**
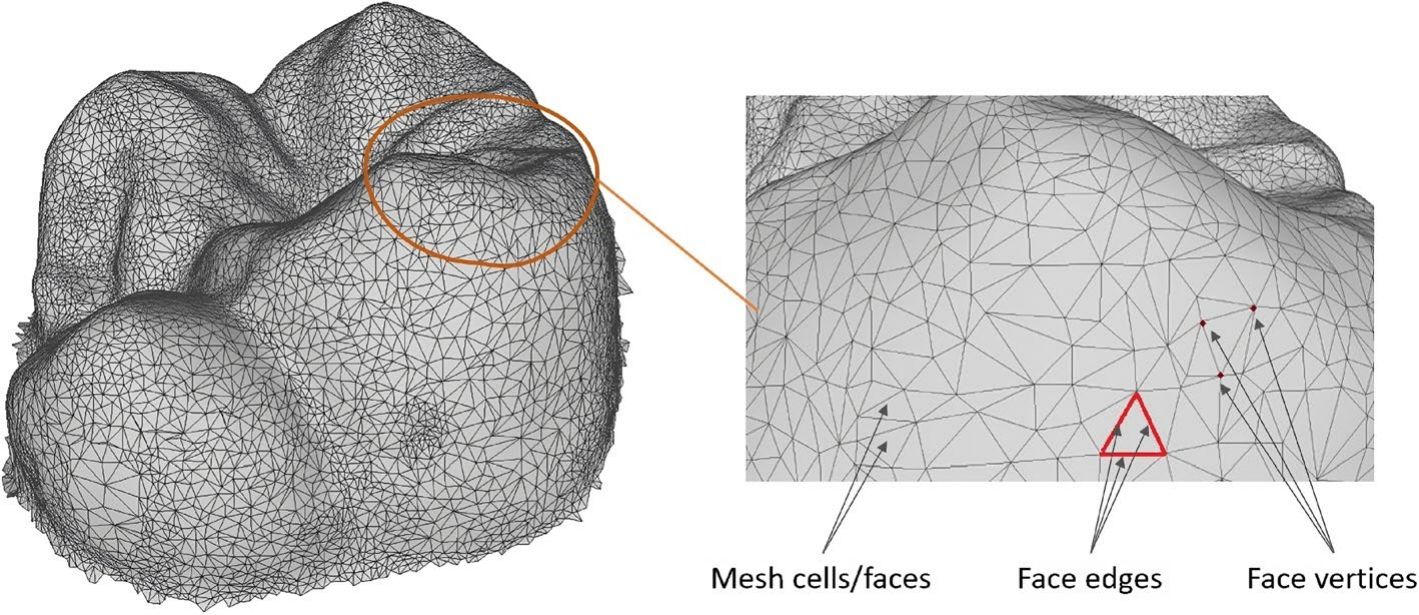
Illustration of 3D mesh structure

A 3D triangular mesh comprises vertices, edges, and faces that define the shape of a 3D object as illustrated in Fig. 1, a triangular mesh is mathematically expressed by:

1 $$\begin{aligned} M = \left( V,F \right) \end{aligned}$$

Where:

- $$V = \{ \nu _i \mid \nu _i \in \mathbb {R}^{3} \}$$.
- $$F = \left\{ f_i \mid f_i \in \left\{ 1,2,\ldots ,\left| V \right| ^3 \right\} \right\}$$.

The vertices **(***V***)** are points in 3D space, the edges connect these points, and the triangular faces **(***F***)** fill the spaces between edges to form a continuous surface **(***M***)**. This structure allows for the detailed modeling of complex surfaces and geometries, making 3D meshes ideal for accurately representing anatomical structures, such as teeth, in dental models. The mesh can capture subtle curves and intricate features of tooth anatomy, which are crucial for precise diagnostics and treatment planning in dentistry. Additionally, 3D meshes are highly versatile and can be manipulated computationally for various applications, including visualization, simulation, and analysis. Their ability to provide detailed and accurate spatial information makes 3D meshes a foundational tool in fields requiring high precision and detailed modeling, such as medical imaging, dental forensics.

### Architecture

**Fig. 2:**
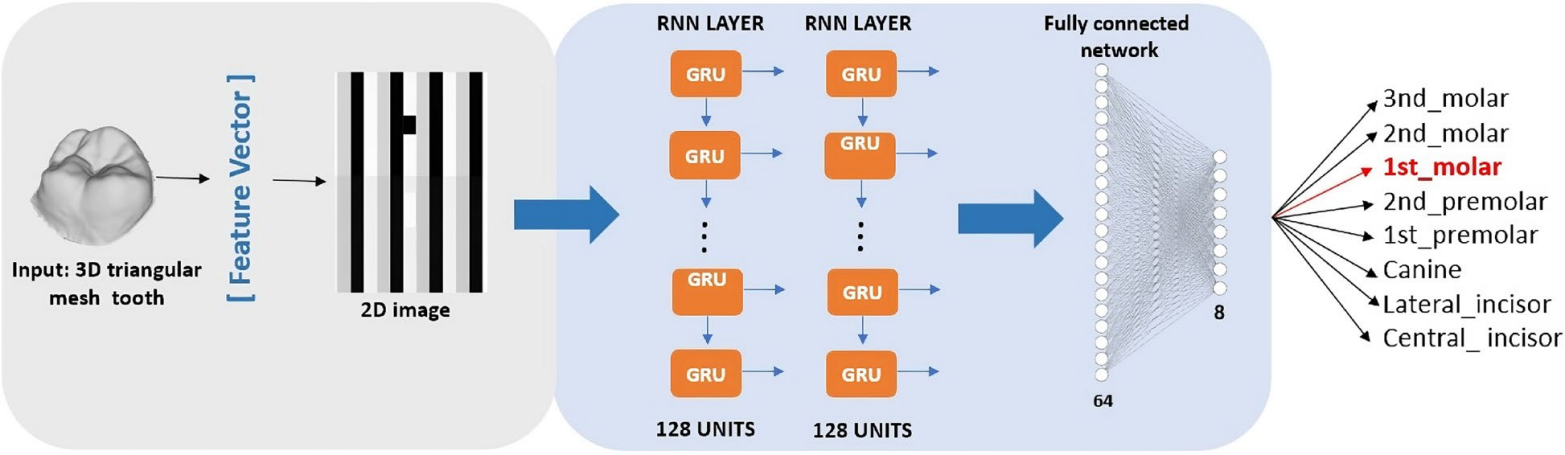
Illustration of the structure of our approach. Our network takes raw 3D mesh data as input, extract specific features, and transform it into 2D image, and processes it through deep learning network

In this study, we present a novel deep learning approach for classifying 3D teeth by extracting features from 3D triangular mesh objects. Our method capitalizes on the geometric intricacies of these dental objects, treating the triangles as time-influenced sequences. This perspective enables us to employ recurrent neural networks, which significantly enhance the accuracy of our classification as Fig. 2 shows.

Our method accepts 3D triangular tooth objects as input. The process begins by extracting representative features from each triangular face of the 3D tooth mesh. Specifically, we extract the vertex coordinates of each triangle and the triangle center, which collectively provide a holistic representation of the mesh by encapsulating both the local geometry and spatial relationships. This approach enables a richer and more precise encoding of the 3D shape. Once the features are extracted, we convert the feature vectors into a 2D grayscale image format. Each row of this image represents a sequence that can be processed by GRU-based neural networks. This transformation effectively bridges the gap between the 3D geometric properties of the tooth and a format that facilitates sequential learning. The 2D image preserves the spatial and geometric relationships of the 3D structure, enabling the GRUs to model both local and global patterns effectively. The transformed 2D feature image is then passed through two layers of recurrent neural networks, each consisting of 128 Gated Recurrent Units (GRUs). GRUs are particularly suited for this task due to their efficient architecture, which balances simplicity and performance. Unlike LSTMs, which have additional gating mechanisms, GRUs streamline the process by combining the forget and input gates into a single update gate. This simplicity reduces computational overhead while retaining the ability to manage sequential dependencies and capture long-term relationships. This capability is especially important in modeling the complex spatial arrangements inherent in the vertices and face centers, which encode intricate geometric variations between different tooth types. In our experiments, GRUs consistently outperformed other architectures, including standard convolutional and LSTM neural networks. CNNs, while effective at capturing local spatial patterns, struggled to model the sequential dependencies required to understand the overall 3D structure of the dental models. LSTMs, although powerful in sequential data processing, introduced unnecessary complexity for this task, leading to overfitting and increased training times due to the limited dataset size. GRUs, in contrast, efficiently captured the anatomical structure of the teeth by focusing on relevant sequential dependencies, enabling the model to generalize better and achieve superior classification accuracy. A more detailed comparison of these models is provided in discussion section (“Discussion” section).

After the GRU layers, the output is fed into two fully connected layers. The first fully connected layer contains 64 neurons, which helps in progressively reducing the dimensionality of the data, filtering out redundant information while retaining the most important features. As the data passes through these layers, complex transformations are applied to facilitate accurate classification. The number of neurons in the final output layer varies depending on the classification scenario: 4, 8, or 16 neurons, corresponding to the number of tooth types being classified. More details about these classification scenarios can be found in the dataset “Dataset” section.

Our architecture employs the sparse categorical cross-entropy loss function, which is well-suited for multi-class classification problems. We utilize the Adam optimizer with a learning rate of 0.0005 to effectively update the network’s weights and biases, ensuring that the model converges quickly while minimizing classification error. This combination of loss function and optimizer has been shown to enhance training stability and improve overall performance.

## Experiments

### Dataset

**Fig. 3:**
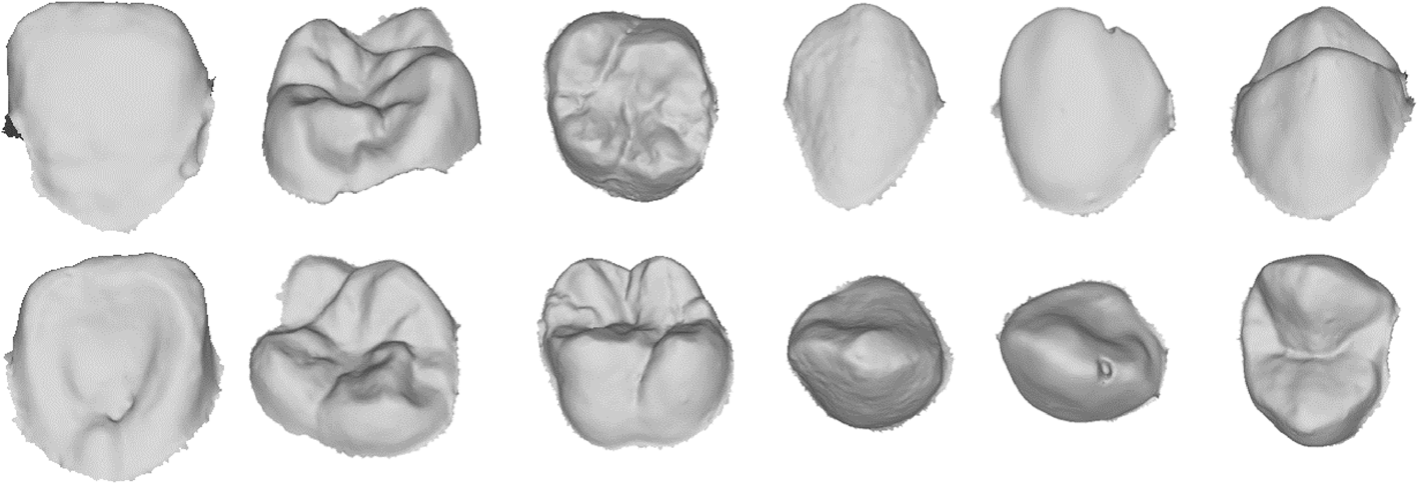
Illustration of examples of 3D teeth in our dataset

The dataset utilized in this study comprises raw maxillary teeth surfaces (Fig. 3), directly acquired and reconstructed by a 3D intraoral scanner. This data collection involved 28 individuals, resulting in 16 distinct tooth types, and yielding a total of 448 tooth objects. Each tooth surface initially contained approximately 100,000 to 150,000 triangular cells. To facilitate processing while preserving the original topology, these surfaces were simplified to 900 triangles per tooth using the quadrilateral method [24]. Ethical approval for this study was obtained, and informed consent was secured from all participants prior to data collection. To safeguard participant privacy and confidentiality, all data were preprocessed and anonymized. We adhered to established ethical standards throughout the data collection, processing, and usage phases, ensuring compliance with research integrity and privacy regulations.

2 $$\begin{aligned} & V' = (R(\alpha ) . V^t)^t \nonumber \\ & \text {Where } R(\alpha ) = \left[ \begin{array}{ccc} \mathrm{cos}^2 \alpha & \mathrm{cos}\ \alpha (\sin ^2 \alpha - \sin \alpha ) & \mathrm{cos}^2 \alpha \sin \alpha + \sin ^2 \alpha \\ \mathrm{cos}\ \alpha \sin \alpha & \sin ^3 \alpha + \mathrm{cos}^2 \alpha & \mathrm{cos}\ \alpha (\sin ^2 \alpha - \sin \alpha ) \\ -\sin \alpha & \sin \alpha \ \mathrm{cos}\ \alpha & \mathrm{cos}^2 \alpha \end{array}\right] \nonumber \\ & - V^t \textit{ is the original vertices and } \varvec{\alpha } \textit{ is the rotation angles} \nonumber \\ & - \varvec{\alpha } \textit{ takes random values between 0 and 360 to generate random rotation angles } \nonumber \\ & - R(\alpha ) \textit{ generates the } [\varvec{\alpha }_x, \varvec{\alpha }_y, \varvec{\alpha }_z] \textit{ that will be applied respectively to x,y and z} \nonumber \\ & \quad \quad \textit{ coordinates of each vertex. } \nonumber \\ & - V' \textit{ represents the new rotated object } \end{aligned}$$

To enhance the robustness and variability of the dataset, extensive data augmentation techniques were applied. By applying random translations and rotations on each tooth object using Eqs. 2 and 3 respectively to simulate various natural orientations and positions. This process generated 6 additional variations for each original tooth object, significantly increasing the dataset’s size and diversity. In total, the augmented dataset comprised 2688 3D tooth objects.

3 $$\begin{aligned} \left[ \begin{array}{c} \nu _{x}^{'} \\ \nu _{y}^{'} \\ \nu _{z}^{'} \\ 1 \end{array}\right] = \left[ \begin{array}{cccc} 1 & 0 & 0 & t_x \\ 0 & 1 & 0 & t_y \\ 0 & 0 & 1 & t_z \\ 0 & 0 & 0 & 1 \end{array}\right] \left[ \begin{array}{c} \nu _{x} \\ \nu _{y} \\ \nu _{z} \\ 1 \end{array}\right] \end{aligned}$$

Where $\nu _x,\nu _y,\nu _z$ are the original vertex coordinates, and $t_x,t_y,t_z$ represent the translation values for the x, y, and z axes, respectively, with each translation value being a random number between 0 and 90.

To thoroughly assess the effectiveness of our approach, we designed three distinct versions of the dataset, each tailored to different classification challenges. This allowed us to rigorously evaluate our model’s ability to handle varying levels of complexity:

- The first version contained all 16 unique tooth types, offering a fine-grained classification task with each class representing a specific tooth.
- The second version contained the teeth organized into 8 broader categories: 3rd molar, 2nd molar, 1 st molar, 2nd premolar, 1 st premolar, canine, lateral incisor, and central incisor.
- The third version contained the dataset divided into 4 major categories: molars, premolars, incisors, and canines.

This multi-version dataset design underscores the comprehensive nature of our study, allowing us to demonstrate the adaptability and robustness of our deep learning model across various levels of classification granularity. Such diversity in the dataset is crucial for demonstrating the robustness and versatility of our method in practical scenarios like dental diagnostics and forensic applications.

During the training process, the dataset was randomly split into training and testing subsets. Specifically, 1881 tooth objects were allocated for training, while the remaining 807 objects were reserved for testing the model. This random split ensures a robust evaluation of the model’s performance and its ability to generalize to unseen data.

### Results

The performance of our proposed 3D teeth classification approach was evaluated and compared against several state-of-the-art methods as Table 1 shows. Our approach demonstrated superior accuracy across different datasets and classification tasks, highlighting its robustness and effectiveness.

**Table 1:** Our dental classification results compared to the state-of-art methods

Scenario\Accuracy	16 Categories	8 Categories	4 Categories
Method			
Iftinca et al. [10]	-	75%	-
Li et al. [1]	-	-	87%
Li et al. [14]	90.7%	-	-
Miki et al. [11]	-	91.21%	-
**Ours**	**98.01%**	**98.88%**	**99.75%**

To provide context, we reference the results achieved by other researchers in similar studies. Iftinca et al. [10] reported an accuracy of 75% on an 8-class dataset using CNNs with the Adam optimizer on 2D images. Despite the effectiveness of CNNs in image processing, the use of 2D images inherently limits the ability to capture the full spatial geometry of teeth, leading to lower classification accuracy. Li et al. [1] employed a seven-layer deep CNN with max pooling and global average pooling on a 4-class 2D CBCT image dataset, achieving an accuracy of 87%. While this method effectively captures and processes image features, it does not fully leverage the 3D information available in dental scans. In a later work Li et al. [14] applied a CNN with a ConvNeXt-S backbone for feature extraction on multi- 2D images of isolated teeth, resulting in an accuracy of 90.7% for tooth identification in 16 classes. This method improves classification by using advanced feature extraction techniques, but still relies on 2D image data. Miki et al. [11] achieved an accuracy of 91.21% on an 8-class dataset using convolutional neural networks (CNNs) applied to cone-beam computed tomography (CBCT) images. This approach leverages the detailed anatomical information provided by CBCT but remains limited by the complexity and variability of 3D tooth structures in 2D projections.

**Table 2:** Performance metrics for each individual tooth type using our proposed architecture on the 16-category dataset version (L: Left, R: Right)

Tooth	Central Incisor (L)	Lateral Incisor (L)	Canine (L)	1st Premolar (L)	2nd Premolar (L)	1st Molar (L)	2nd Molar (L)	3rd Molar (L)	Central Incisor (R)	Lateral Incisor (R)	Canine (R)	1st Premolar (R)	2nd Premolar (R)	1st Molar (R)	2nd Molar (R)	3rd Molar (R)
Metric																
Precision	100%	100%	100%	100%	97.27%	98.31%	91.31%	88.26%	100%	100%	100%	100%	99.75%	96.95%	93.34%	87.22%
Recall	100%	100%	100%	99.75%	98.31%	98.31%	94.01%	89.29%	100%	96.33%	100%	99.75%	100%	97.27%	94.27%	86.38%
F1-score	100%	100%	100%	99.87%	97.79%	98.31%	92.64%	88.77%	100%	98.13%	100%	99.88%	99.87%	97.11%	93.80%	86.70%

In comparison, our proposed approach, which utilizes 3D to 2D transformation combined with representative features vector and subsequent processing through RGU and fully connected networks, yielded superior results.For the 16-class dataset, our method achieved an accuracy of **98.01%**. For the 8-category dataset, our approach reached an accuracy of **98.88%**. For the 4-class dataset, our method attained an impressive accuracy of **99.75%**. The transformation of 3D features into a 2D image format based on the feature vector allowed for the effective application of RGU and fully connected networks, capturing the complex spatial relationships and subtle variations between different tooth types, thus demonstrating the robustness and precision of our classification system.

In addition, we present in Tables 2 and 3 a detailed evaluation of the classification performance of the proposed method across the different versions of the dataset. The tables gives, precision, recall, and F1-score metrics for a full evaluation of the model’s performance. In all dataset versions, the our proposed architecture achieves consistently high scores.

**Table 3:** Performance metrics of our proposed architecture on the 8-category (a) and 4-category (b) dataset versions

	(a) 8-Category Dataset Version								(b) 4-Category Dataset Version			
Tooth	Central Incisors	Lateral Incisors	Canines	1st Premolars	2nd Premolars	1 st Molars	2nd Molars	3rd Molars	Lateral Incisor	Canines	Premolars	Molars
Metric												
Precision	100%	100%	100%	100%	98.03%	100%	98.15%	93.26%	100%	100%	99.31%	97.41%
Recall	100%	100%	100%	99.56%	99.13%	97.12%	97.15%	96.13%	100%	99.75%	99.15%	97.38%
F1-score	100%	100%	100%	99.82%	98.57%	98.53%	97.64%	94.67%	100%	99.87%	99.22%	97.39%

In the 16-class dataset version, classification performance is robust for most tooth classes (Table 2). However, 2nd and 3rd molars exhibit slightly lower precision and recall. The performance decrease was mainly attributed to their morphological similarity with adjacent tooth types, making it hard for the model to discriminate between them. Moreover, third molars, having relatively low representation in the dataset, provided fewer training samples for the model, limiting its capacity to generalize adequately for this class. In the 8-category dataset version (Table 3-(a)), where teeth are grouped into broader categories, the performance remains high, with a slight improvement in overall classification consistency. The consolidation of similar teeth into fewer categories mitigates the issue of inter-class similarity, thereby enhancing classification reliability. Similarly, in the 4-category dataset version, where teeth are further grouped into incisors, canines, premolars, and molars (Table 3-(b)), the method demonstrates near-perfect identification results across all categories, underscoring its robustness in broader classification tasks.

Overall, the proposed approach maintains high classification accuracy across all dataset verions, demonstrating its adaptability to different levels of tooth categorization. The observed variations in performance for certain classes are primarily due to data distribution and inherent structural similarities between adjacent teeth, yet the method remains highly effective in distinguishing between them.

### Discussion

To evaluate the impact of different neural network architectures and feature extraction elements, we conducted a comprehensive ablation study. Our results, presented in Tables 4 and 5, reveal several key insights into how each component of the model contributes to overall classification accuracy across the three dataset versions : 16 categories, 8 categories, and 4 categories.

We first observed that CNNs (convolutional neural networks) achieved the lowest performance, with accuracies of 76.45%, 81.41%, and 90.33% for the 16, 8, and 4-category datasets, respectively. This underperformance stems from the inherent limitations of CNNs in handling sequential data. While CNNs excel at extracting spatial features, they struggle to capture the temporal relationships and structural dependencies within the sequential feature data derived from 3D tooth objects. To address this, we experimented with recurrent architectures like vanilla RNN and LSTM, which are designed to capture temporal dependencies in sequential data. The vanilla RNN demonstrated significant improvements over CNNs, yielding 90.08% accuracy on the 16-category dataset and reaching up to 98.02% on the 4-class dataset. LSTM, which mitigates vanishing gradients through memory gates, further improved accuracy to 93.92% for the 16-category classification. However, its complexity sometimes led to overfitting, particularly in fine-grained tasks like the 16-class dataset. Thus, while both RNN and LSTM outperformed CNNs, their performance still fell short of expectations due to challenges in managing long-term dependencies and generalizing to complex dental structures.

**Table 4:** Our dental classification results compared to state-of-the-art methods

Scenario\Accuracy	16 Categories	8 Categories	4 Categories
Network type			
CNN	76.45%	81.41%	90.33%
Vanilla RNN	90.08%	93.30%	98.02%
LSTM	93.92%	96.28%	98.14%
**GRU**	**98.01%**	**98.88%**	**99.75%**

The most significant improvements were observed with GRU networks. GRUs, being simpler than LSTMs but still capable of managing temporal dependencies, provided a balanced solution that outperformed both RNNs and LSTMs across all scenarios. As seen in Table 4, GRUs achieved 98.01% accuracy for the 16-category dataset, 98.88% for the 8-category dataset, and 99.75% for the 4-class dataset. This highlights the GRU’s superior ability to generalize across different levels of classification granularity while maintaining high accuracy. Additionally, the number of GRU layers and units played a crucial role in the model performance. We found that while increasing the number of GRU layers or units initially improved accuracy, further increases led to overfitting.

**Table 5:** Comparative performance of different neural network types in the proposed approach

Scenario\Accuracy	16 Categories	8 Categories	4 Categories
Features			
GRU with only vertices	94.29%	96.09%	98.63%
GRU with only triangles center	94.79%	97.69%	98.76%
**GRU[vertices + triangle centers]**	**98.01%**	**98.88%**	**99.75%**

The choice of features also had a critical effect on model performance. Initially, we experimented with using only the vertices’ coordinates for feature extraction, inspired by previous works that implemented only vertices [25–27]. However, this yielded suboptimal results, as shown in Table 5, where GRU with only vertex features resulted in accuracies of 94.29%, 96.09%, and 98.63% for the 16, 8, and 4-class datasets, respectively. The vertices alone, while capturing the corners of each triangle, failed to provide enough geometric context about the overall surface structure of the 3D object, leading to a loss of crucial spatial information. Alternatively, by using only the triangular face centers, there was a slight improvement, with accuracies of 94.79%, 97.69%, and 98.76%. However, the most substantial gain was achieved when we combined both vertices’ coordinates and the triangular face centers. This combination provided the model with a more holistic representation of the mesh by improving the spatial and structural feature representation of the teeth. The results of this combined approach, as indicated in Table 5, reached accuracies of 98.01%, 98.%, and 99.75%, demonstrating the importance of using both sets of features for capturing the complex 3D structure of dental objects.

In addressing the clinical relevance of our findings, the proposed method demonstrates significant potential for implementation in forensic dentistry, particularly in scenarios where traditional identification methods are unavailable or ineffective. In mass disaster situations-such as tsunamis, plane crashes, or fires...-teeth often remain intact due to their exceptional resilience, making them a reliable and critical source for identification. Our approach, which achieves 98.01% accuracy in classifying 16 tooth types, offers a powerful tool for automating the identification process by analyzing 3D dental models. This automation significantly reduces the time and expertise required by forensic odontologists, enabling faster and more efficient victim identification. This capability is especially vital in cases involving decomposed, disfigured, or fragmented remains, where dental records frequently serve as the primary means of identification. Furthermore, the method can be seamlessly integrated into digital forensic workflows, facilitating rapid and accurate matching of antemortem dental records with post-mortem data. By leveraging the rich 3D anatomical information captured in dental scans, our approach not only enhances identification accuracy but also streamlines the entire forensic investigation process. These advancements underscore the method’s potential to transform forensic dentistry, offering a scalable, precise, and efficient solution for real-world applications in disaster victim identification (DVI) and beyond.

## Conclusion

In this study, we presented a novel deep learning approach for 3D teeth classification, effectively addressing critical challenges in dental image analysis. Our method involves extracting features from each triangular face of the 3D object, transforming these features into 2D grayscale images, and treating each row as a sequence for input into a recurrent neural network. This strategy captures the sequential nature of the data, leading to significant improvements in classification accuracy across various scenarios: 16 categories, 8 categories, and 4 categories. By outperforming existing methods, our approach demonstrates both efficiency and accuracy in tooth type identification. The robustness of our method lies in its ability to model complex spatial relationships within 3D tooth structures, while also being scalable across different levels of classification detail. However, several limitations should be acknowledged. The dataset used in this study consists of 448 tooth objects, which, although substantial, may still limit the model’s generalizability. Although data augmentation techniques were applied to increase the dataset size, this approach could lead to overfitting, particularly due to the limited number of original samples. This risk of overfitting may affect the model’s performance when applied to new, unseen data in real-world scenarios. Moreover, while our approach demonstrates promising results, the effectiveness of the model in diverse populations and clinical settings has yet to be fully validated. Future work will focus on expanding the dataset size, improving model accuracy by exploring more complex architectures, and incorporating additional features to enhance the robustness of the method. Additionally, efforts will be directed toward integrating this approach into existing forensic workflows, validating its performance across diverse demographic datasets, and addressing potential challenges related to data acquisition and processing in field conditions. Addressing these limitations- particularly by increasing the dataset’s diversity, and ensuring adaptability to real-world forensic scenarios- will further strengthen the contribution of this research to the field of automated dental analysis and forensic dentistry.

## Data Availability

The datasets used and/or analysed during the current study are available from the corresponding author on reasonable request.
